# Accuracy of clinical risk factor-based models as a screening test for detecting gestational diabetes mellitus in a low-resource setting

**DOI:** 10.20945/2359-4292-2026-0020

**Published:** 2026-03-02

**Authors:** Olayinka Comfort Senbanjo, Fatimat Motunrayo Akinlusi, Kabiru Afolarin Rabiu

**Affiliations:** 1 Department of Obstetrics and Gynaecology, Lagos State University Teaching Hospital, No 1-5 Oba Akinjobi Way, GRA, Ikeja, Lagos State, Nigeria; 2 Department of Obstetrics and Gynaecology, Lagos State University College of Medicine, Ikeja, Lagos State, Nigeria

**Keywords:** Accuracy, gestational diabetes mellitus, clinical risk factor-based models, screening, low resource setting

## Abstract

**Objective:**

Screening and diagnosing gestational diabetes mellitus (GDM) usually requires a 2-hour, 75 g oral glucose tolerance test (OGTT), which can be challenging for both patients and healthcare systems. Alternative clinical risk factor-based models have been suggested but have not been extensively tested, particularly in low-resource countries. This study aimed to evaluate the accuracy of these risk factor-based models as screening tools.

**Subject and methods:**

This prospective cohort study involved 400 consenting pregnant women receiving antenatal care in Lagos, Nigeria. Participants were evaluated for GDM risk using three clinical models and underwent universal screening and diagnosis at 24 to 28 weeks with a single-step, 2-hour 75g OGTT, using IADPSG/WHO criteria. The Receiver Operating Characteristic (ROC) curve was used to assess the accuracy of the risk factor-based models.

**Results:**

The mean age of the subjects was 31.0 ± 5.3 years. The prevalence of GDM, according to the IADPSG/WHO 2013 criteria, was 19.0%. Using the clinical risk score models developed by Naylor and cols., Caliskan and cols., and Phaloprakarn and cols., positive risk scores for GDM were found in 85%, 67.3%, and 93.8% of subjects, respectively. The sensitivity, specificity, and accuracy of these models ranged from 71.1% to 96.1%, 6.7% to 33.6%, and 23.8% to 40.8%, respectively. However, the negative predictive values were relatively high, ranging from 83.2% to 88%.

**Conclusion:**

The clinical risk factor-based prediction models evaluated in this study may effectively identify women at low risk for GDM who can be exempted from the 2-hour OGTT.

## INTRODUCTION

Globally, the prevalence of gestational diabetes mellitus (GDM) ranges from 1% to 28%, depending on the location, characteristics of the studied population, and the diagnostic criteria used ^(1)^. More than 80% of the burden of GDM is found in lowand middle-income countries, and it is speculated to contribute significantly to the high maternal and infant mortality rates in these countries ^(2)^. In the United States ^(3)^, recent estimates show that GDM complicates up to 9% of all pregnancies, while the mean prevalence of GDM for countries in Europe is 5.4% ^(4)^. In Africa, a systematic review of studies on GDM reported a pooled prevalence of about 14%, contributing significantly to the total global burden of gestational diabetes ^(5)^. In Nigeria, the prevalence of GDM ranges between 0.3% and 35.9% ^(6,7)^. It is reported that Nigeria has the highest prevalence in Africa, and GDM is one of the five most common medical conditions complicating pregnancy in women attending maternal and child health care facilities in Nigeria ^(7-9)^.

Every pregnant woman needs to be screened for gestational diabetes mellitus (GDM) because pregnancy provides a crucial opportunity to identify and treat this condition, ensuring favorable pregnancy outcomes and preventing the progression to type 2 diabetes mellitus and other serious complications.

Currently, the oral glucose tolerance test (OGTT) is considered the gold standard for confirming GDM diagnosis. ^(10)^. While this method enables healthcare providers to identify nearly all pregnant women with GDM, it has some limitations, such as poor reproducibility, long waiting times, and patient discomfort, including nausea and vomiting in some cases ^(11)^. Additionally, this method may be impractical in lowand middle-income countries due to cost constraints, resulting in lower acceptance of the test ^(2,11,12)^. As a result, several professional organizations have suggested using alternative tests, including clinically based risk factors, as screening tools for GDM ^(2,11,13)^. However, it is important to note that while screening based on risk factors has been proposed by several professional organizations, many other international organizations, such as the American Diabetes Association ^(14)^ and the Brazilian Society of Diabetes ^(15)^, advocate for universal biochemical screening for all pregnant women. This is because using only risk-factor-based approaches tends to miss a significant number of cases.

In sub-Saharan African countries, many health facilities relied on a checklist of GDM-associated risk factors to select pregnant women who should undergo the diagnostic test using the OGTT ^(8,16,17)^. However, GDM in this high-risk population remained underdiagnosed ^(8,17)^. The use of risk factors as a screening test and the diagnostic accuracy of the risk factors are unclear either when used separately or collectively as a checklist. It is also unknown whether the use of the risk factor assessment is preferable to plasma glucose measurement as a general screening strategy for GDM at the first contact. There are established clinical risk factor-based models that have been developed and advocated for use in some countries, such as Naylor and cols. in Canada ^(18)^, Caliskan in Turkey ^(19)^, and Phaloprakarn in Bangladesh ^(20)^. The widespread applicability of these models has not been tested, especially in lowand middle-income countries.

The aim of this study was to determine the prevalence of GDM and to evaluate the diagnostic accuracy of these established clinical risk factor-based models for the detection of GDM in Lagos, Nigeria.

## SUBJECTS AND METHODS

### Study setting

The study was conducted at the antenatal clinic of the Institute of Maternal and Child Health, Ayinke House, Lagos State University Teaching Hospital (LASUTH), Ikeja, Lagos State, Southwestern Nigeria. LASUTH is one of two teaching hospitals in Lagos State. It is located in Ikeja Local Government Area and is owned by the Lagos State Government. The hospital functions as a training center for resident doctors and provides healthcare for residents of Lagos State and surrounding areas. The Institute of Maternal and Child Health, Ayinke House, is managed by the Department of Obstetrics and Gynecology. The obstetrics services are provided through the antenatal clinic (ANC) and the emergency room. These services care for both booked and unbooked pregnant women. The ANC accepts and cares for all pregnant women who choose to register at LASUTH. The ANC operates every day except weekends, serving an average of 30 patients daily. It is staffed by approximately four Consultant Obstetricians and Gynecologists, five resident doctors, and four nurses.

### Study design

This study was a prospective, hospital-based, cohort study of all consecutive consenting pregnant women at the gestational age of 24 weeks to 28 weeks.

### Ethical approval and consent to participate

The study was conducted in full accordance with current relevant laws and international agreements. Approval to carry out this research was granted by the Health Research and Ethics Committee of Lagos State University Teaching Hospital, Ikeja, Lagos State, with approval number LREC/06/10/1403. Written informed consent was obtained from all participants who agreed to take part in the study.

### Inclusion criteria

These are pregnant women who booked for antenatal care and had their booking weight and height measured and recorded at gestational age less than or equal to 24 weeks and with singleton pregnancy.

### Exclusion criteria

Pregnant women with a history of pre-gestational diabetes mellitus, on drugs that can affect glycaemic profile, such as steroids and beta-agonists, multiple pregnancies, and unwilling to participate in the study were excluded from the study.

### Sample size

The minimum number of subjects’ ‘n’ required for the study was estimated from the formula:


$$n=z^{2}p(1-p)÷d^{2}$$

Where ‘n’ is the desired sample size,

‘z’ is the critical value and in a two-tailed test, it is equal to 1.96.

‘p’ is the prevalence of gestational diabetes from previous studies in Nigeria. Previous studies in Nigeria gave the prevalence of gestational diabetes within ranges of 0.5% - 35.9%. For this study, a prevalence of 35.9% by Onyenekwe and cols. was used ^(7)^.

‘d’ is the absolute sampling error that can be tolerated. In this study, it was fixed at 5 percent

Therefore, the minimum sample size ‘n’ = 1.96^2^ x 0.359 x (1 - 0.359) ÷ 0.05^2^ = 353.8 which is approximately 354. Taking into consideration a possible attrition rate of 10% among pregnant women, the minimum sample size for this study was 389.4. This sample size was rounded up to 400.

### Selection of study participants and test procedures

Prior to the commencement of the study, the researcher informed the doctors and nurses in the Department of Obstetrics and Gynaecology about the research and the recruitment protocol. The researcher also trained three research assistants on the use of the study proforma and the different measurements to be taken with hands-on demonstration to ensure standardization.

Data were obtained from all consented pregnant women using a purposely designed interviewer-administered questionnaire and from the information in Naylor and cols. ^(18)^, Caliskan and cols. ^(19)^, and Phaloprakarn and cols. risk scoring models ^(20)^. Information collected includes socio-demographic characteristics, obstetric history (such as parity, previous miscarriage, history of GDM, and history of perinatal death), and family history of diabetes mellitus. The families were assigned a socio-economic class using the method recommended by Ogunlesi and cols. ^(21)^. Those with mean scores of 1 and 2 were further classified as upper class, those with mean scores of 3 as middle class, and those with mean scores of 4 and 5 as falling into the lower social class. Dating of the pregnancy and gestational age estimation was based on the first day of the last menstrual period in women with a 28-day menstrual cycle, compared with the woman’s fundal height. The researcher used the earliest ultrasound (i.e., a scan done at less than or equal to 20 weeks of gestation) if the woman was unsure of her date. The weight and height of all subjects measured at gestational ages less than or equal to 24 weeks were recorded. Random blood glucose measurements were performed in all selected subjects at their first contact. Afterward, all participants were requested to return to the antenatal clinic fasting after a week for an Oral Glucose Tolerance Test (OGTT) to confirm the presence of GDM. The test was conducted between the 24th and 28th week of gestation.

A venous blood sample was taken from the participants to perform random blood glucose tests using an analyzer based on the enzymatic oxidase-peroxidase method of glucose measurement. After ensuring aseptic technique, about three milliliters of venous blood were drawn from a vein in the antecubital fossa with a 21G needle butterfly device equipped with a safety system. The blood was collected into a vacutainer tube containing sodium fluoride, a glycolytic inhibitor, and kept on ice from the time of phlebotomy until it was delivered to the laboratory. Each blood sample was centrifuged within two to four hours of collection to obtain plasma. The plasma samples were stored at -20°C and pooled together to determine plasma glucose concentration. The resulting value was recorded in the participant’s study proforma.

All study participants were required to arrive early in the morning for an oral glucose tolerance test after fasting for approximately 8 to 12 hours overnight. Each patient was called the day before the test to remind her to fast. A 75-gram glucose load was prepared by dissolving 75 grams of anhydrous glucose in 250 to 300 ml of clean water. Participants were advised to drink the liquid as quickly as comfortable and to remain relaxed, avoiding vigorous activity during the test. Venous blood for glucose measurement was collected as described above, and samples were analyzed using the enzymatic reaction of the oxidase-peroxidase method for glucose estimation.

The first sample collected prior to the glucose load is the fasting plasma glucose, while the second and third blood samples shall be taken at 1 hour and 2 hours, respectively, after the glucose load.

### Diagnostic cut-offs

Gestational diabetes mellitus in the participants was diagnosed using the criteria from clinical risk factor-based models and plasma glucose levels according to the International Association of Diabetes and Pregnancy Study Groups/World Health Organization (IADPSG/WHO 2013) guidelines ^(10,12)^. GDM prevalence as determined by the IADPSG/WHO 2013 criteria was used as the reference standard.

#### Naylor and cols. clinical risk score ^(18)^

The screening score is based on clinical variables such as age, body mass index (BMI), and ethnicity. Using these variables, women were assigned a clinical risk score, with a maximum possible score of 10 points. Women with scores of 0-1 were categorized as low risk, while those with scores of 2-3 and scores higher than 3 were categorized as intermediate and high risk, respectively. For this study, women with a score of 0-1 were considered negative for GDM, while those with scores of 2-10 were considered positive for GDM.

#### Caliskan and cols. clinical risk score ^(19)^

The screening score was based on clinical variables such as age, BMI, family history of diabetes mellitus, a prior pregnancy with a baby weighing more than 4000g, and previous adverse pregnancy outcomes (defined as any of the following: recurrent spontaneous abortions, fetal anomalies despite a normal karyotype, or prior unexplained in utero fetal death at a gestational age over 20 weeks). A score of one was assigned for each of these five variables, with a maximum possible score of five. A risk score greater than 2 is considered sufficient to diagnose GDM.

#### Phaloprakarn and cols. clinical risk score ^(20)^

This risk factor-based model relies on clinical risk variables such as age over 35 years, BMI above 27 kg/m^2^, a first-degree relative with type 2 diabetes mellitus or a personal history of GDM, prior delivery of a macrosomic infant, or previous adverse pregnancy outcomes (more than 2 miscarriages, congenital malformations, or stillbirth). The risk score is calculated using the equation: 6 times the woman’s age plus 11 times her BMI plus 109 if there is a family history of diabetes in a first-degree relative, plus 42 if she previously delivered a baby weighing more than 4000 g, plus 49 if there has been an adverse pregnancy outcome such as two or more abortions, congenital malformations, or stillbirth. A score greater than 380 indicates a positive screen for GDM.

### Data analysis

Data entry and analysis were conducted using SPSS version 24 software. Descriptive statistics such as frequency, percentages, means, standard deviations, and the corresponding 95% CI were used to summarize the variables. The sensitivity, specificity, positive predictive value, and negative predictive value of the clinical risk factor-based models were calculated by identifying the total number of women with true positive, true negative, false positive, and false negative results relative to the gold standard 2-hour 75g OGTT. A receiver-operating characteristic (ROC) curve was plotted, and the area under the curve (AUC) was calculated to assess the overall performance of the clinical risk factor-based models. A p-value of less than 0.05 was considered statistically significant for all tests.

## RESULTS

### Socio-demographic characteristics of the study participants

The socio-demographic characteristics of the participants are shown in **Table 1**. Their age ranges from 18-51 years with a mean age of 31.0 ± 5.3 years. Most (93.8%) of the participants were in the age group 21-40 years. They are predominantly of the Yoruba tribe (70%) and the modal parity was nullipara (53%). Three hundred and forty-five (86.3%) participants belonged to the high socio-economic class (social class 1).

**Table 1:** Socio-demographic and anthropometric characteristics of the study population

Parameters	Frequency	Percentages
Age (years) ≤ 20 21-30 31-40 > 40	5 196 179 20	1.2 49.0 44.8 5.0
Tribe Yoruba Hausa Ibo Others	280 1 61 58	70.0 0.2 15.3 14.5
Parity 0 1 2-4 ≥ 5	212 98 81 9	53.0 24.5 20.3 2.2
Occupation Professional/technical/managerial Clerical, sales and services, skilled manual Unskilled manual, farmer, and other	145 232 23	36.2 58.0 5.8
Highest level of education No formal education Primary Secondary Tertiary	0 6 57 337	0.0 1.5 14.2 84.3
Husband’s Occupation Professional/technical/managerial Clerical, sales and services, skilled manual Unskilled manual, farmer, and other	258 136 6	64.5 34.0 1.5
Husband’s highest level of education No formal education Primary Secondary Tertiary	1 4 49 346	0.2 1.0 12.3 86.5
Social class 1 2 3	345 50 5	86.25 12.5 1.25
^*^Weight (kg) ^*^Height (Meter)	72.9 ± 16.1 1.62 ± 0.065	
Body mass index <18.5 18.5 -24.9 25-29.9 ≥ 30	10 137 126 127	2.5 34.3 31.5 31.8

* Values are means (SD).

### Obstetric characteristics of the study participants

**Table 2** shows the obstetric history of the study participants. Thirty (7.5%) participants gave a history of previous delivery of a macrosomic baby, 22 (5.5%) had a previous history of stillbirth, 58 (14.5%) had a previous history of miscarriage, 23 (5.8%) had a previous history of GDM, 10 (2.5%) had a previous history of congenital anomaly and only one (0.25%) had a previous history of polyhydramnios. Fifteen (3.8%) of the participants had the conception of the index pregnancy by artificial reproductive technology.

**Table 2:** Obstetric characteristics of the study population

Parameters	Frequency	Percentages
Previous macrosomic infant ≥ 4000g No Yes	370 30	92.5 7.5
Previous unexplained stillbirth No Yes	378 22	94.5 5.5
Previous abortion or miscarriage No Yes	338 58	84.5 14.5
Previous history of GDM No Yes	377 23	94.3 5.7
Index pregnancy by artificial reproductive technology No Yes	385 15	96.2 3.8
Fetal gender of index pregnancy Male Female Don’t know	89 51 260	22.3 12.8 65.0
Previous history of congenital anomaly No Yes	390 10	97.5 2.5
Unexplained polyhydramnios No Yes	399 1	99.25 0.25

### Prevalence of GDM using the various diagnostic criteria

**Table 3** shows the prevalence of GDM based on various diagnostic and screening criteria. According to the IADPSG/WHO 2013 guideline, the prevalence of GDM based on the “gold standard” 2-hour OGTT was 19.0%. The prevalence based on FPG was 10.5%, whereas none of the participants met the criteria for GDM based on 1-hour OGTT. A total of 76 participants met the criteria of either 2-hour OGTT and or FPG giving a prevalence of 19.0%.

**Table 3:** Prevalence of GDM using various diagnostic criteria

Test and reference cut-off values	Mean (SD)	95% CI	No (%)
IADPSG/WHO 2013 guideline Fasting plasma glucose > 92 mg/dL (5.1 mmol/L) 1-hour OGTT > 180 mg/dL (10 mmol/L) 2-hour OGTT > 153 mg/dL (8.5 mmol/L) FBS and or 2-hour OGTT	79.1 (10.7) 112.6 (14.5) 110.4 (31.6) -	78.1-80.2 111.2-114.1 107.3-113.5 -	42 (10.5) 0 (0.0) 52 (13.0) 76 (19.0)
Naylor and cols. clinical risk score 0-1 (Low risk) 2-3 (Intermediate risk) >3 (High risk)	- - -	- - -	60 (15.0) 182 (45.5) 158 (39.5)
Caliskan and cols. clinical risk score 0-1 (Negative screen) ≥ 2 (Positive screen)	- -	- -	131 (32.8) 269 (67.2)
Phaloprakarn clinical risk score < 380 (Negative screen) ≥ 380 (Positive screen)	- -	- -	25 (6.2) 375 (93.8)

According to the clinical risk score by Naylor and cols., 340 (85%) participants had a risk score that was significant enough to consider diagnostic screening for GDM while the risk score by Caliskan and cols. shows that 269 (67.3%) participants can be considered to have GDM. However, the clinical risk score by Phaloprakarn and cols. shows that 375 (93.8%) participants can be considered to have GDM.

### Accuracy of selected published clinical risk-based scores and fasting plasma glucose for the diagnosis of GDM

**Table 4** shows the diagnostic accuracy of selected published clinical risk scores for diagnosing GDM, using the IADPSG/WHO 2013 diagnostic criteria as the gold standard. All tested clinical risk scores exhibited low specificity, ranging from 6.7% to 33.6%, and a positive predictive value (PPV) between 19.5% and 20.1%. However, the sensitivity and negative predictive value (NPV) were comparatively higher, with sensitivity ranging from 71.1% to 96.1% and NPV from 83.2% to 88%. The highest accuracy was observed with the Caliskan clinical risk score screening test, which achieved an accuracy of 40.8%. In contrast, the fasting plasma glucose (FPG) test demonstrated low sensitivity (53.9%) but exhibited high specificity (98.8%), PPV (91.1%), NPV (90.1%, and accuracy (90.3%).

**Table 4:** Accuracy of selected published clinical risk scores and fasting plasma glucose in the diagnosis of GDM using IADPG/WHO 2013 diagnostic criteria as reference standard

Parameter	TP	FN	FP	TN	Sensitivity (95% CI)	Specificity (95% CI)	PPV (95% CI)	NPV (95% CI)	Accuracy (95% CI)
Naylor and cols.	67	9	273	51	88.2 (80.9-95.5)	15.7 (11.7-19.7)	19.7 (15.5-23.9)	85.0 (76.0-94.0)	29.5 (25.0-34.0)
Caliskan and cols.	54	22	215	109	71.1 (60.9-81.3)	33.6 (28.5-38.7)	20.1 (15.3-24.9)	83.2 (76.8-89.6)	40.8 (36.0-46.0)
Phaloprakarn and cols.	73	3	302	22	96.1 (91.8-100.0)	6.7 (4.1-9.5)	19.5 (15.5-23.5)	88.0 (75.3-100.0)	23.8 (19.6-28.0)
FPG (> 5.1 mmol/L)	41	35	4	320	53.9 42.3-65.2	98.9 96.5-99.6	91.9 78.6-96.8	90.1 86.4-93.0	90.3 86.7-93.0

**Figure 1:**
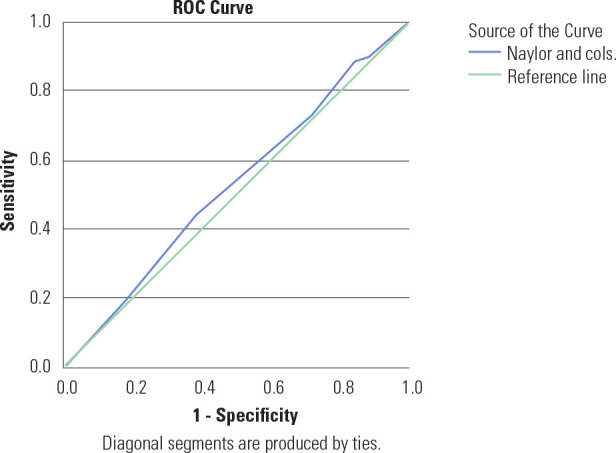
Receiver operating characteristic curves showing performance of Naylor and cols. clinical risk score in predicting GDM.

**Figure 2:**
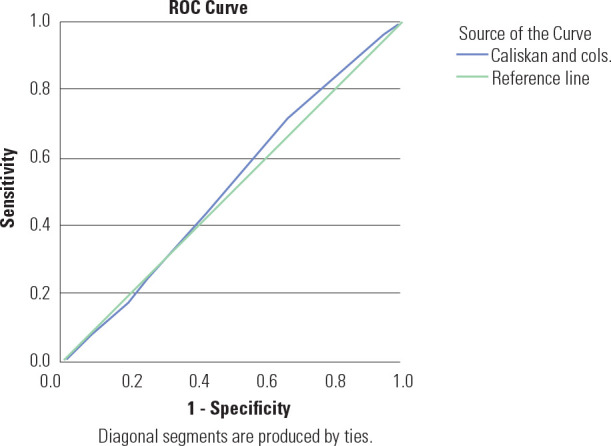
Receiver operating characteristic curves showing performance of Caliskan and cols. clinical risk score in predicting GDM.

**Figure 3:**
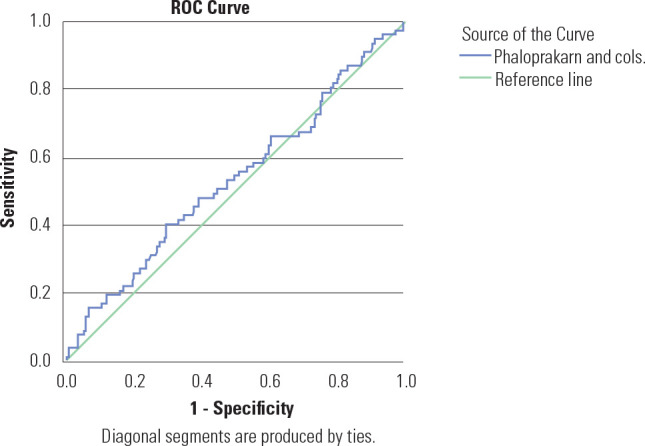
Receiver operating characteristic curves showing performance of Phaloprakarn and cols. clinical risk score in predicting GDM.

**Figure 4:**
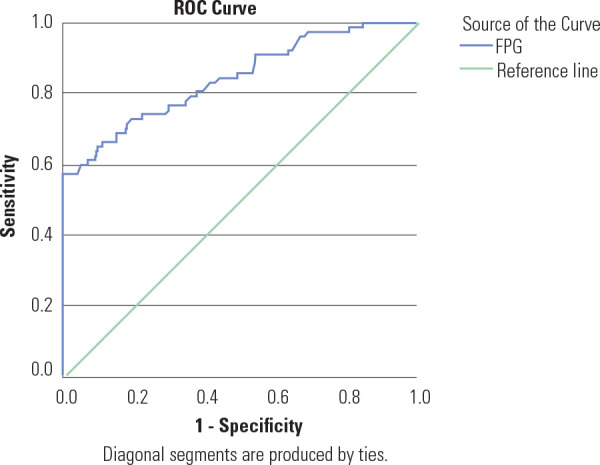
Receiver operating characteristic curves showing the performance of FPG in predicting GDM.

Figures 1-3 illustrate the ROC curves for the clinical risk factor-based models in our population, which had an AUC ranging from 51.6% to 52.9%, indicating unsatisfactory discriminatory capacity. The ROC curve for FPG showed an AUC of 84.1%, reflecting a good performance for screening of GDM (**Figure 4**).

Figure 1 shows the ROC curves for the Naylor and cols. clinical risk score indicating the performance of the test with reference to IADPSG diagnostic criteria. The Area Under the Curve (AUC) was 0.526 (95% CI 0.453-0.598) suggesting that the clinical risk score is non-discriminatory.

Figure 2 shows the ROC curves for the Caliskan and cols. clinical risk score indicating the performance of the test with reference to IADPSG diagnostic criteria. The Area Under the Curve (AUC) was 0.516 (95% CI 0.446-0.586), suggesting that the clinical risk score is non-discriminatory.

Figure 3 shows the ROC curves for the Phaloprakarn and cols. clinical risk score indicating the performance of the test with reference to IADPSG diagnostic criteria. The Area Under the Curve (AUC) was 0.529 (95% CI 0.455-0.603), suggesting that the clinical risk score is non-discriminatory.

Figure 4 shows the ROC curves for FPG, indicating the test’s performance relative to IADPSG diagnostic criteria. The Area Under the Curve (AUC) was 0.841 (95% CI 0.786-0.897), suggesting that the test has a good performance for screening of GDM.

## DISCUSSION

The prevalence of gestational diabetes mellitus (GDM) among women in Lagos, based on the IADPSG/WHO 2013 criteria, was found to be 19%. This figure is significantly higher than the range of 1.5% to 11.5% reported in earlier studies conducted in Lagos between 1987 and 2004 ^(22-24)^. These earlier studies employed different diagnostic criteria, which may account for the observed different prevalence rates. This finding is in keeping with the study by Goyal and cols., which reveals a marked variation in the prevalence of GDM, accompanied by a significant degree of disagreement among different diagnostic criteria ^(25)^. Additionally, the prevalence of GDM in this study surpasses the 4.9% reported in Ibadan by Fawole and cols. about 10 years ago, despite their study only including women at high risk for GDM ^(17)^. The observed increase in GDM rates may be attributed to the global rise in obesity and overweight conditions over the years.

Interestingly, the prevalence of GDM in this study aligns closely with the 24% found in a recent study at the Lagos University Teaching Hospital, although the methods used for glucose assays in that study were not specified ^(26)^. Conversely, a study by Onyenekwe and cols. ^(7)^, conducted in southeastern Nigeria, reported a GDM prevalence of 35.9%. This may have been an overestimation due to the reliance on glucometers, which could compromise the accuracy of plasma glucose measurements, as well as the fact that the oral glucose tolerance test (OGTT) was only conducted on those with risk factors for GDM ^(7)^.

While there is a substantial body of literature on GDM prevalence in Nigeria, the varying results can largely be attributed to different diagnostic criteria and glucose assay methods used by researchers. When compared to studies outside Nigeria that utilized the IADPSG diagnostic criteria, the prevalence of GDM in Lagos was notably higher than figures from the United States (7.6%), Ireland (7.2%), and Turkey (14.5%), yet lower than the 34.9% reported in Punjab, India, and 38.6% in Kuala Lumpur, Malaysia ^(27-31)^.

This aligns with previous findings that indicate lower GDM prevalence rates in North America and Europe compared to those in sub-Saharan African countries. In contrast, countries in the Middle East, North Africa, and Southeast Asia demonstrate the highest global prevalence of GDM. Notably, the prevalence in this study was similar to the 18.3% reported in the Marrakech and Safi districts of Morocco ^(32)^. This trend may signify a growing burden of GDM in Nigeria. It is crucial, therefore, to ensure that all pregnant women are screened for GDM and receive timely diagnoses to facilitate appropriate treatment for those affected.

There are indications that combining clinical risk factors to develop a clinical prediction model may be more effective in accurately detecting women with GDM than using each risk factor individually for screening ^(33)^. In this study, all the evaluated models based on clinical risk factors showed high sensitivity, low specificity, and poor overall accuracy in identifying GDM. This suggests that while these models are good at detecting many cases of GDM, they could also be useful for screening to identify women who are unlikely to have GDM.

Instructively, the methodology for the risk factor-based scoring models used a two-step GDM screening strategy, which differs from the IADPSG/WHO guidelines and the practice in the index study. The goal of each model is not to replace challenge-based glucose tolerance testing but to exclude low-risk women from further testing. If our study participants had been selectively screened based on Naylor, Caliskan, and Phaloprakarn risk scores, 15%, 32.8%, and 6.3%, respectively, would not have undergone diagnostic testing. Of this total, 85%, 83.2%, and 88%, respectively, would be negative if universal screening with a diagnostic test was performed. The high negative predictive values highlight that risk factor-based models could be useful tools for excluding GDM and reducing the need for OGTT screening. However, due to low PPV, any woman testing positive on these models should undergo a formal 75 g 2-hour OGTT to confirm the diagnosis. This approach can lower costs for patients and improve healthcare system efficiency, especially in low-resource settings.

The models demonstrated high sensitivity but low specificity and poor accuracy, which is consistent with the study by Adam and Reeder ^(34)^. They evaluated eight clinical prediction models in the South African population, including the three models analyzed in this study, and found all eight models performed poorly in detecting GDM. The area under the ROC curve for the three models tested in this study ranged from 51.6% to 52.9%, similar to the 51.8% to 59.4% range found in South Africa. These findings suggest that these models are ineffective in differentiating between individuals with GDM and those without it, which makes them non-discriminatory. This limitation is significant, particularly in a country like Nigeria, where resources may be scarce and accurate screening for GDM is essential. With this limitation, there is clear evidence that these specific models are not suitable for the Nigerian population without significant modification. A recent study from Southern India that assessed the utility of clinical parameters as a screening tool for GDM reported a high sensitivity of 90.4% and a specificity of 32.1% ^(35)^. This model incorporated several clinical parameters along with biochemical factors such as triglycerides, which may help explain the slightly higher sensitivity and specificity observed. However, the area under the ROC curve was only 68%, indicating marginal discriminative power. These findings indicate that clinical risk factor-based prediction models serve primarily as effective rule-out tests for screening women for GDM.

The diagnostic accuracy of the models tested in this study is lower than the 73.3% to 83.2% reported for the derivation populations of these models. Among the three clinical prediction models evaluated, the Caliskan model demonstrated the highest accuracy. The Caliskan risk score successfully identified or predicted approximately 41% of cases with GDM, while the Naylor and cols. and Phaloprakarn and cols. risk scores identified about 30% and 24%, respectively. The low discriminatory power of these tests may be attributed to their derivation from a different population, as well as the use of a selective screening approach and diagnostic criteria that differ from the IADPSG guidelines in developing these scoring systems. Beyond the influence of ethnicity and the use of different diagnostic criteria in the derivation of the models, the risk factors for GDM and their respective weights can vary significantly across Caucasian, Asian, and African populations. The models that were tested were not specifically developed or validated for a West African population, which may explain their low accuracy in this context. Additionally, the current study used early-pregnancy BMI instead of pre-pregnancy BMI. Early pregnancy weight is often slightly higher than pre-pregnancy weight, which would systematically raise the BMI. This may inflate the models’ sensitivity while further reducing their specificity, especially for those that depend heavily on this metric.

In Nigeria, the guidelines for the management of GDM were developed by a team of endocrinologists only ^(36)^. The recommendations include risk assessment at booking, a one-step (75-g OGTT), or a two-step method (50-g GCT with 100-g OGTT) using Carpenter and Coustan criteria for diagnosis. The Carpenter and Coustan criteria define specific fasting and postprandial plasma glucose levels after a 100-gram oral glucose tolerance test, which, if exceeded, indicate GDM ^(37)^. Despite the availability of this guideline on GDM, the practice varies across obstetric units in Nigeria. This may be because the guideline is not precise in its documentation and appears impractical. It is suggested that the risk models may be considered by obstetricians to screen out patients going for the diagnostic test. However, aside from saving cost and time, we must bear in mind the false negative cases and the known benefits of treatment. It is important to highlight that the review of guidelines for managing GDM in Nigeria, based on established, practical, and accepted international standards, is long overdue. Moreover, with the findings in this study showing that the FPG test has good performance for screening of GDM. In Brazil, the Society of Diabetes 2025 guideline, explicitly recommend an alternative diagnostic pathway starting with an FPG at the first prenatal visit, followed by a repeat FPG between 24-28 weeks if the initial test is normal ^(17)^. Therefore, further research into the accuracy of biochemical screening parameters, such as the FPG test or the 50g glucose challenge, as second-line screening tests to identify false-negative cases detected by risk models, warrants further consideration.

This study has some limitations. First, the study used weight and height measured in early pregnancy for the calculation of body mass index, unlike the risk factor-based models assessed in this study which used pregravid BMI of participants for computing their risk score. This may have affected the sensitivity and specificity of these clinical risk-based scoring models. In lowand middle-income countries, it is difficult to get a record of pre-pregnancy weight as most women do not go for preconception consultations in health facilities. Nonetheless, the influence of pregnancy on weight status at the early stage is considered minimal, and as such, measurement of weight and height and their association to compute BMI remains a valuable tool for the assessment of nutritional status in pregnancy. Second, most of the study subjects were of the high socio-economic class, and this may have an influence on the prevalence of GDM. Lastly, this study was carried out in a tertiary health facility that receives high-risk referrals. Therefore, the reported GDM prevalence of 19.0% and the performance of the screening models may not be generalizable to primary care or rural settings, where such a screening tool is most needed. In conclusion, the burden of GDM is high in Lagos, Nigeria. This is similar to other resource-poor nations where available health facilities are grossly inadequate to take care of the existing problem of infectious diseases and the rising epidemics of non-communicable diseases. The clinical risk factor-based predictive models examined in this study could be utilized to identify a low-risk population of women who should be exempted from the cumbersome OGTT. Furthermore, efforts should focus on developing a cost-effective screening tool with an optimal cut-off value that is both highly sensitive and more specific for detecting GDM, particularly in low-resource settings.

## Data Availability

datasets related to this article will be avail-able upon request to the corresponding author.
